# Community Pharmacies Face Critical Sustainability Challenges in the United States: Academic Pharmacy Can Help

**DOI:** 10.3390/pharmacy14020054

**Published:** 2026-03-29

**Authors:** Karl M. Hess, Peter Lim

**Affiliations:** School of Pharmacy, Chapman University, Irvine, CA 92618, USA

**Keywords:** community pharmacy, reimbursement, renumeration, pharmacy deserts, business, management, entrepreneurship, pharmacy rotations, IPPEs, APPEs

## Abstract

Community pharmacies in the United States (US) face an increasingly unsustainable future due to declining third-party reimbursement (remuneration) and ongoing cash flow challenges following the elimination of retroactive direct and indirect remuneration (DIR) fees. These pressures have contributed to widespread pharmacy closures, the emergence of pharmacy deserts, and reduced access to care for millions of patients. Despite these challenges, community pharmacy remains the most common employment setting for pharmacy school graduates in the US. However, currently required community pharmacy Advanced Pharmacy Practice Experience (APPE) student rotations may offer limited exposure to business, management, and entrepreneurial activities, potentially leaving students underprepared for practice in this setting. US colleges and schools of pharmacy are uniquely positioned to address this gap by partnering with their community pharmacy APPE rotation sites to intentionally integrate business- and practice-focused knowledge, skills, and attitudes (KSAs) into the APPE. Equipping students with these KSAs may enhance early career readiness while also supporting the financial sustainability of US community pharmacies through the development of innovative, revenue-generating services. These efforts further align with the 2025 Accreditation Council for Pharmacy Education (ACPE) Standards and may help advance the profession. Future research should examine optimal community pharmacy APPE structures, models, and assessment strategies to maximize student preparedness and long-term community pharmacy sustainability.

## 1. Background

Community pharmacies are vital hubs where the public can quickly and easily access healthcare services and receive face-to-face expert pharmacist care to help ensure the safe and effective use of medications [1,2]. While there is some variation in how far away individuals may live from a community pharmacy, overall, 89.9% of the US population lives within five miles of a chain or independent community pharmacy [3]. Despite the widespread availability of community pharmacies and the public’s reliance on them for their healthcare needs, the current business model is unfortunately not sustainable, as revenue is primarily tied to third-party reimbursement (remuneration) for medication dispensing, where such rates have steadily declined over time. The unintended consequence of the Centers for Medicare and Medicaid Services eliminating retroactive direct and indirect remuneration (DIR) fees in 2024 was the creation of a “DIR cliff,” which has further worsened pharmacies’ financial stability. As a result, all pharmacy prescription insurance fees are now taken at the point of sale when a medication is picked up at the pharmacy. While this makes prescription dispensing fees more transparent, their shift to the point of sale unfortunately reduces the revenue pharmacies have available, which can worsen cash flow by limiting the immediate funds the pharmacy has available for operations or other investments. Third-party reimbursement for non-dispensing patient care services also remains limited, further impacting the financial viability of community pharmacies as patients continue to rely on their services as pharmacies try to remain in business [4].

The impact of these reimbursement challenges can be observed in pharmacy closure data. From 2010 to 2021, 29.4% of pharmacies closed in the US, and from 2018 to 2021, forty-one states experienced a net loss of pharmacies. Independent pharmacies were also more at risk for closure than chain pharmacies (38.9% versus 21.9%, respectively), and these pharmacies were more likely to be in predominantly Black or Latinx neighborhoods or in areas with higher poverty or uninsured rates of 20% or more as compared to their chain counterparts. Independent pharmacies were also two times more likely to be the only pharmacy in the neighborhood. As a result, independent pharmacies were more than two times more likely to close compared to chain stores [5]. Previous studies have also confirmed similar results. From 2009 to 2015, one in eight pharmacies closed in the US, affecting independent pharmacies and low-income neighborhoods more, and particularly those in urban areas [6]. Similarly, in rural settings, 16.1% of independent pharmacies closed from 2003 to 2018 [7]. Pharmacies that were excluded from the Medicare Part D insurance preferred prescription network also faced a significantly higher risk of closure (70% to 350%), with independent pharmacies and those in low-income, Black, and Latinx neighborhoods disproportionately affected [8]. To better visualize the ongoing and widespread problem of pharmacy closures, the American Association of Colleges of Pharmacy (AACP), in partnership with the Academia-Community Transformation (ACT) Collaborative, maintains an interactive map of pharmacy closures in the US, with external links to media reports about such closures [9].

## 2. Objectives and Methods

The purpose of this paper is to highlight the challenges currently facing community pharmacies in the US and to offer solutions that may help to alleviate these challenges utilizing colleges and schools of pharmacy. PubMed was searched for published articles on pharmacy deserts, community pharmacy reimbursement, and business, entrepreneurship, and/or management education in colleges and schools of pharmacy in the United States. Published articles from outside of the United States were not used for the purpose of this paper. As a result, information from other countries is not included, and comparison to the United States was not undertaken. Findings from these searches are provided below.

## 3. Results and Discussion

### 3.1. Impact of Pharmacy Closures

The closure of community pharmacies ultimately leads to the formation of pharmacy deserts, which are defined as areas wherein it takes longer for an individual to travel to a pharmacy than to a supermarket [10]. It is currently estimated that 17.7% of Americans (representing 57.1 million individuals) live in a pharmacy desert. Furthermore, an additional 8.9% Americans (representing 28.9 million individuals) rely on a single “keystone pharmacy”, defined as a pharmacy whose closure would result in a pharmacy desert, in their community. Independent pharmacies are also more likely than chains to be a keystone pharmacy, and rural settings tend to be more dependent on these keystone pharmacies [10]. The sustainability of community pharmacy in the US therefore needs to be addressed, since pharmacy closures reduce patient access to healthcare services and may even be indicative of a looming public health crisis in the US [10,11]. In at least one report, pharmacy closures were found to be associated with declines in patient adherence to cardiovascular medications, supporting the impact that pharmacy closures can have on public health [12].

### 3.2. Better Preparing Students to Enter the Community Pharmacy Workforce

Student pharmacists in the US increasingly perceive community pharmacy as a less attractive career path after starting pharmacy school [13]. This declining interest, coupled with a decrease in pharmacist employment in community pharmacies from 60% in 2014 to 54% in 2022, may highlight a trend where pharmacists are shifting to other areas of employment [14]. Despite this, community pharmacies remain the top employment sector (59.1%) for pharmacists in the US [15]. To prepare students for this area of practice, community pharmacy introductory and advanced pharmacy practice experience student rotations (IPPEs and APPEs), which typically occur during the last year of a student’s education, currently focus on medication distribution and patient counseling activities as defined by the Accreditation Council for Pharmacy Education (ACPE) Standards for pharmacy education in the US, which require students to gain exposure to the “continuity of patient care and acute, chronic, and wellness promoting services” [16]. The literature has identified a critical need for better incorporating entrepreneurship into pharmacy education to prepare students for the evolving healthcare landscape and to drive practice advancement for the purposes of sustainability. This includes developing effective teaching methods, student interest in pharmacy ownership, and essential skills for innovative practice [17]. This is further supported by a report that found 63% of students strongly agreed that their required community pharmacy APPE site provided them with dispensing skills, compared to only 36% that strongly agreed it provided them with management skills. This may be the result of community pharmacy IPPEs focusing on fundamental pharmacy activities while community pharmacy APPEs focus on preparing students to be staff-level pharmacists [18,19]. This lack of exposure to entrepreneurship could ultimately hinder students’ preparedness for and success in community pharmacy practice and may impact the sustainability of community pharmacies.

Interestingly, ACPE has removed the terms innovation and entrepreneurship from Appendix 1 of its updated Standards 2025—two elements critical for preparing students as leaders in community pharmacy practice and for the sustainability of the profession. At the same time, the terms diagnosing and prescribing were added, and they have been included to ensure consistency with the training necessary to practice in states that allow full scope practice authority [16,20]. This raises a key concern: how can the Academy expect students to be prepared for advanced roles such as diagnosing and prescribing if innovation and entrepreneurship, the drivers of such advancements, are no longer emphasized or included in our accreditation standards, especially as pharmacies continue to close due to reimbursement issues?

Therefore, to help prepare students for the challenges that currently exist in community pharmacy, high-quality community pharmacy IPPE and APPE student rotations must be intentionally designed and educationally robust. As part of this effort, entrepreneurship-related knowledge, skills, and attitudes (KSAs) could be incorporated into experiential learning evaluations to assess student progression towards practice readiness in the community pharmacy setting, with an emphasis on the long-term professional sustainability of community pharmacy. However, there is currently no consensus within US pharmacy education regarding which entrepreneurship KSAs could or should be included or elevated to the level of educational standards (or that they even should be). Therefore, to help inform curricular development in the absence of such consensus, colleges and schools of pharmacy could look to previous research conducted by Mattingly et al. Mattingly’s research surveyed successful entrepreneurs, pharmacy faculty members, students, and other community leaders to identify key KSAs needed in pharmacy entrepreneurship (or “Pharmapreneurship”, as coined by these researchers) to help guide course design and assessment [21]. By thoughtfully integrating selected entrepreneurship-related KSAs into community pharmacy experiential settings and assessing them as part of mid-point and final evaluations, colleges and schools of pharmacy can help ensure graduates are not only clinically competent to provide advanced patient care services but also equipped to serve as effective stewards of the profession who are prepared to contribute to the long-term sustainability of community pharmacy practice moving forward.

### 3.3. Experiential Education Examples That Could Help Guide US Colleges and Schools of Pharmacy

There are various examples in the literature of innovative community pharmacy APPEs that could serve as examples towards these aims. In one such example of a four-week community pharmacy APPE elective student rotation, students were utilized as facilitators for community pharmacy transformation activities using the Community Pharmacy Enhanced Services (CPESN) framework, which showed positive increases in student understanding of business management following intervention [22]. In another, students, who were primarily on a leadership APPE rotation, developed protocols for selected disease states, such as uncomplicated urinary tract infections, tobacco cessation, Lyme disease, cold and canker sores, and pink eye, which were subsequently vetted by pharmacy experts, shared with community pharmacists, and then incorporated into community pharmacy practices [23]. Other such examples include the Partners for Promotion Program, where students completed a ten-month longitudinal community pharmacy APPE rotation to help implement and expand advanced patient care services with community pharmacy preceptors; a practice transformation APPE rotation which paired students with community sites to help implement and evaluate pharmacy services; and a community pharmacy APPE rotation where students were utilized as practice transformation coaches to help implement patient centered care models at community pharmacy sites [24,25,26].

While the above are examples of specific experiences at different colleges and schools of pharmacy in the US, each illustrates how students can help identify and implement meaningful changes through a unique and actionable solution that mirrors the core processes of business development and entrepreneurship and helps to develop high-quality IPPEs and APPEs student rotations that can better prepare students for real-world community pharmacy practice. In these examples, students are not only engaged in practice transformation but are also learning how to assess operational challenges, design service innovations, and implement improvements—skills that are directly aligned with entrepreneurial thinking, innovation, and business acumen. These experiences help to expose students to opportunity recognition, strategic planning, resource management, and value creation, which are essential components of transforming community pharmacy practice. Through real-world involvement in practice transformation efforts, student pharmacists can gain hands-on experience that can be applied to future careers as staff pharmacists, pharmacy managers, or pharmacy owners. General examples of different community pharmacy rotation models have been included in Table 1 below for pharmacy colleges and schools to consider as these experiences further develop.

Regardless of the specific model developed by a college or school, some important considerations before launching such experiences include site readiness and its support capabilities for hosting students (e.g., access to pharmacy data, buy-in from ownership, pharmacist and staff support) and whether a faculty member could serve as the primary preceptor at that site to help offset any workload or capacity issues at the pharmacy. By outsourcing pharmacy innovation and development opportunities to students under the supervision of a faculty member, the pharmacy benefits from receiving additional assistance and experience at no cost. Faculty, in turn, are incentivized to create a high-quality rotation experience for their IPPE and APPE rotation students, while their students benefit from real-world experiences in business-oriented problem solving and innovation, which benefits the pharmacy site. With the proper structure, incentives, and defined roles for the student, preceptor, and site, this model has the potential to develop future pharmacy entrepreneurs and innovators capable of contributing to, advancing, and helping to sustain community pharmacy practice for future generations.

Not all community pharmacy sites are positioned or willing to support high-quality student experiential education experiences that extend beyond medication dispensing, as some settings may choose to prioritize their operational efficiencies or staffing needs over student learning. This may limit the student’s opportunity for meaningful exposure to or experience in business, management, or entrepreneurial activities at the site. When experiential sites are unable or unwilling to meet defined educational standards, continued use of these sites for community pharmacy IPPE and APPE rotations should be reevaluated. Experiential education should remain learner-centered; sites that may primarily utilize students to support their workflow needs can undermine student educational outcomes and may inadvertently reinforce unsustainable practice models rather than preparing students to address them.

## 4. Conclusions

The continual reimbursement challenges community pharmacies in the US face and the rate of pharmacy closures in this country call for immediate action from US colleges and schools of pharmacy. If colleges and schools of pharmacy are concerned about the advancement of community pharmacy practice and its sustainability, they should partner with willing community pharmacy experiential rotation sites to help support and advance their practice transformation efforts. Such action will not only help colleges and schools of pharmacy address the practice management domain of Appendix 1 in the 2025 ACPE Pharmacy Accreditation Standards but will also support community sites in developing new patient care services and creating potential revenue streams for them. Ultimately, this will help create and reinforce high-quality community pharmacy IPPE and APPE student rotations, which could help improve the students’ overall community pharmacy experiential learning and better prepare them for the challenges that await them as licensed community pharmacy practitioners. Preparing students to function within otherwise unsustainable practice models that only serve the immediate operational needs of the site while deprioritizing student education is insufficient and may only contribute to the status quo. Future research should focus on identifying the ideal structures or models for community pharmacy IPPE and APPE student rotations to maximize impact on student preparedness to ensure that graduates are equipped as skilled practitioners as well as innovative and entrepreneurial leaders capable of navigating and improving the landscape of community pharmacy practice to ensure its future sustainability.

## Figures and Tables

**Table 1:** Examples of high-quality community pharmacy rotation models.

Example Experiential Model	Core Student Activities	Value to Practice Site	Educational and Other Outcomes	Key Considerations
Practice Transformation	Workflow analysis Service gap identification Implementation planning	Operational improvements Groundwork for new services	Student development of business acumen/skills Creation of patient care protocols	Site readiness (site personnel and resources) Faculty oversight to reduce site burden
Enhance Service Development	Design and pilot billable services Document workflows	Potential revenue diversification Service sustainability	Service design Student reimbursement literacy	Access to site data Payer variability
Consultancy	Needs assessment surveying Benchmarking Standard operating procedure development	Expert-level assistance at no cost	Student-applied problem solving	Clear role definitions for student, faculty member, site personnel Faculty oversight to reduce site burden
Leadership-Focused	Staff training tools Audit Preparation	Improved operational efficiency	Student development of management and leadership competencies	Buy-in from pharmacy site leadership
Hybrid	Structured deliverables based on hybrid components	Reduced workload for site preceptors and staff	Integrate specific business and entrepreneurship components into the rotation	Faculty time allocation Institutional support

## Data Availability

The original contributions presented in this study are included in the article. Further inquiries can be directed to the corresponding author.
